# A Retrospective Study of Imaging of Invasive Rhino-Orbital-Cerebral Mucormycosis in the COVID-19 Pandemic in a Tertiary Care Center

**DOI:** 10.7759/cureus.61629

**Published:** 2024-06-04

**Authors:** Pushpa Ranjan, Vinod Kumar, Neetu Sinha, Aditya Abhishek Jaiswal, Deepak Kumar, Sanjay K Suman

**Affiliations:** 1 Radiodiagnosis, Indira Gandhi Institute of Medical Sciences, Patna, IND

**Keywords:** retroantral, pansinusitis, intraconal, extraconal, periantral, paranasal sinus, covid -19, fungal, mri, ct

## Abstract

Aim

The study aims to analyze the imaging findings of invasive rhino-orbital-cerebral mucormycosis (ROCM) in patients who had COVID-19.

Materials and methods

This retrospective descriptive study was done on confirmed (culture and histopathology) patients who had a COVID-19 infection. The data was collected from the record section from May 2021 to June 2021. Imaging data were analyzed, and findings were tabulated according to statistical methods.

Results

Radiological evaluation, including CT and MRI, was done in 48 cases. The ethmoid sinus was the most common sinus involved in 60.41% of cases, followed by the maxillary sinus (52.09%). Unilateral pansinusitis was observed in 21 cases (43.75%). Among periantral extensions, retroantral fat involvement was the most common finding, seen in 24 cases (50%). Lamina papyracea and the walls of the maxillary sinus were involved in eight cases (16.67%). A total of 38 cases (79.17%) exhibited involvement of the extraconal compartment, while 32 cases (66.67%) showed involvement of the intraconal compartment. In intracranial involvement, infarct was noted in 13 cases (27%), and cavernous sinus involvement in nine cases (18.75%).

Conclusions

ROCM is a life-threatening fungal infection in immunocompromised patients, especially diabetics. Imaging of ROCM plays a pivotal role in early diagnosis, the extent of disease, surgical planning, prognosis, and the response to treatment. Radiologists must know the imaging features and patterns of extension of ROCM.

## Introduction

COVID-19 was reported as pneumonia in December 2019 in Wuhan, a city in the Hubei Province of China, which was renamed SARS-CoV-2 and was declared by WHO as a global pandemic in March 2020. This pandemic spread worldwide with high morbidity and mortality due to superadded coinfection. It presents with respiratory symptoms of mild to severe pneumonia with multisystem involvement. This infection affected patients with comorbid conditions like diabetes, hypertension, chronic kidney disease, lung disease, and cardiac disease.

The second wave of COVID-19 in India was devastating due to a surge of mucormycosis among patients with COVID-19. Mucormycosis is an opportunistic infection that usually occurs in immunocompromised patients; however, diabetes mellitus is one of the most common predisposing factors. Fulminant progression was common and sometimes led to death in less than a week after presentation. The Government of India asked the states to notify all cases of the so-called “black fungus” under the Epidemic Diseases Act [1].

Rhino-orbital-cerebral mucormycosis (ROCM) in COVID-19 patients was associated with high mortality due to local invasion through vessels and perineural spread. The angioinvasive nature of mucormycosis causes ischemia, with subsequent infarction and necrosis of affected areas that warrant aggressive management. Early diagnosis was based on a high index of suspicion, depending upon clinical features. Imaging plays a very important role in early diagnosis, surgical planning, prognosis, complications, and follow-up. Early diagnosis and immediate surgical intervention, along with antifungal treatment, saved the sight as well as the life of the patient.

ROCM in patients with COVID-19 infection is owing to the presence of a hyperinflammatory state due to overexpression of cytokines, delayed interferon-gamma response, and impairment of cell-mediated immune response due to decreased counts of CD4+ and CD8+ T lymphocytes, which facilitate the development of invasive fungal infection [2-6]. Impaired glycemic control, rampant use of steroids to suppress severe inflammatory syndrome [1], the use of broad-spectrum antibiotics, prolonged hospital stays, oxygen therapy, and ventilatory support were responsible for this superinfection, resulting in high mortality.

The primary objective of this study is to describe the CT and MRI findings of ROCM in patients with COVID-19 infection. Additionally, this study aims to determine the frequency of involvement of different anatomic structures in ROCM, assess the imaging patterns of disease spread, stage the severity of ROCM, and correlate imaging findings with clinical features and comorbidities.

## Materials and methods

Patient selection

This study was conducted at the Indira Gandhi Institute of Medical Sciences, Patna, India. Patients were randomly selected for study from the record section between May 2021 and June 2021. The diagnosis of COVID-19 was documented based on an RT-PCR positive or rapid antigen-positive report of a naso-oropharyngeal swab. Patients selected for the study had a confirmed diagnosis of mucormycosis based on either fungal culture using a potassium hydroxide mount or histopathological confirmation. The study included 48 patients, 14 female and 34 male, with an age range of 21-87 years and a mean age of 49.54 ± 12.8. All patients underwent CT scans with rhino-orbital symptoms, and 26 patients with clinical symptoms related to the brain and orbit underwent MRIs of the brain and/or orbit. Contrast was given to patients after normal renal function. Those patients who did not undergo imaging (CT scan and MRI) in our institute but had a confirmed diagnosis of mucormycosis were not included in the study.

Imaging protocol

A CT scan was done on the Aquilion 64 (TSX-101A) (Toshiba Medical Systems and Aquilion Lightning, Canon Medical Systems, 16-row, 32 slice). Low osmolar non-ionic contrast with an iodine content of 350 mg/mL was given at a dose of 1 mg/mL with a pressure injector. For paranasal sinuses, scans were taken from the top of the hyoid to the top of the frontal sinuses, both in soft tissue and sharp (bone) kernels, at a slice thickness of 1 mm and 210 mm field of view (FOV). An MRI was done on the GE OPTIMA MR360 1.5 Tesla. An MRI of the paranasal sinus, brain, and orbit was done in the axial, coronal, and sagittal plane with conventional sequences T1W, T2W, FLAIR, SWAN, and T1FS with or without contrast at a slice thickness of 3 mm with a 1 mm gap and a 17 cm FOV from the nose tip to the brain stem. An intravenous injection of gadopentetate dimeglumine (0.1 mmol/kg) was given, and images were acquired in the T1FS sequence. Contrast was not given to patients with abnormal renal function. Additional sequences like DWI, ADC, TOF/contrast-enhanced angiogram, and venogram were also taken according to the types of lesions.

Image analysis

Images were analyzed by three radiologists with experience spanning more than 10 years and were blinded to clinical data. They looked for the site and extent of involvement and complications like involvement of the cavernous sinus, including the internal carotid artery, intracranial infarct, fungal abscess, encephalitis, and meningitis. Other associated findings, like bone changes (erosion and destruction) and patterns of enhancement on both CT scans and MRIs, were interpreted.

Statistical analysis

The data entry was done in the Microsoft Excel spreadsheet (Microsoft Corporation, Redmond, WA, USA), and the final analysis was done with the use of IBM SPSS Statistics for Windows, Version 21.0 (Released 2012; IBM Corp., Armonk, NY, USA). The presentation of the categorical variables was done in the form of numbers and percentages (%).

Ethical considerations

The study was approved by the Institutional Ethics Committee of the Indira Gandhi Institute of Medical Sciences, Patna, India, with a waiver of informed consent for the retrospective analysis.

## Results

Demography and clinical background

The age range of patients was from 21 to 87 years, with a mean age of 49.5 ± 12.8. The maximum number of patients (90.5%) were between 41 and 60 years old. Out of 48 patients, 34 (65%) were male and 14 (29.17%) were female.

Among the 48 cases, 43 (89.58%) were associated with type II diabetes mellitus and 14 (29.17%) with hypertension. Other associated comorbidities are given in Table 1.

**Table 1:** Associated comorbid disease distribution.

Associated disease	Frequency	Percentage
No comorbidity	3	6.25%
Type II diabetes mellitus	43	89.58%
Hypertension	14	29.17%
Chronic kidney disease	6	12.50%
Hypothyroidism	4	8.33%
Acute kidney injury	5	10.42%
Hematological malignancy	1	2.08%
Hepatitis B	1	2.08%
Sepsis	1	2.08%
Lung fibrosis	1	2.08%
Anemia	1	2.08%
Glaucoma	1	2.08%

Imaging findings

Sinonasal Imaging

**Figure 1:**
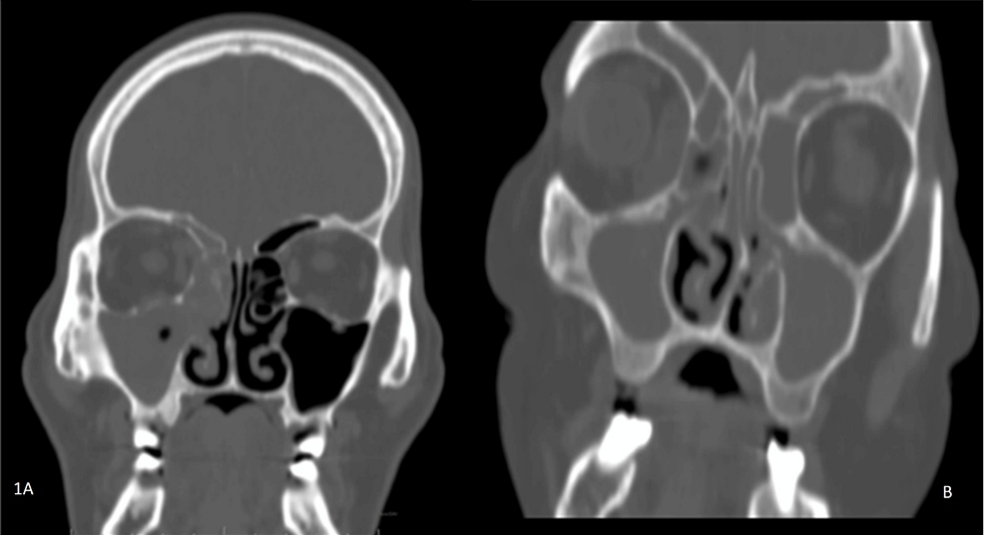
Coronal CT scan images of paranasal sinuses in the bone window depict two male patients aged 48 years and 50 years. (A) Unilateral pansinusitis on the right side shows opacification of the right maxillary sinus, ethmoid sinus, and frontal sinus with the widening of the osteomeatal complex, erosion of the uncinate process, middle turbinate, and bony lamellae of the ethmoid sinus. (B) Bilateral pansinusitis demonstrates opacification of the bilateral maxillary sinus, ethmoid sinus, and frontal sinus.

In the current study, a CT scan showed that the ethmoid sinus was the most common sinus involved in 60 (41%) cases, followed by the maxillary sinus (52.09%). Unilateral pansinusitis was observed in 21 cases (43.75%). The involved sinus may show features of unilateral pansinusitis or bilateral pansinusitis, which usually show complete opacification with soft tissue attenuation along with bone changes as erosion (Figure 1A, 1B). Involvement of other sinuses and surrounding areas is depicted in number and percentage in Table 2 under the heading of paranasal sinuses.

**Table 2:** CT findings distribution.

CT findings	Frequency	Percentage
Paranasal sinuses		
Pansinusitis (unilateral)	21	43.75%
Maxillary sinus (unilateral)	17	35.42%
Maxillary sinus (bilateral)	8	16.67%
Ethmoid sinus (unilateral)	16	33.33%
Ethmoid sinus (bilateral)	13	27.08%
Frontal sinus (unilateral)	14	29.17%
Frontal sinus (bilateral)	5	10.42%
Sphenoid sinus (unilateral)	11	22.92%
Sphenoid sinus (bilateral)	8	16.67%
Preantral/premaxillary area	23	47.92%
Retroantral area	24	50%
Pterygopalatine fossa and surrounding area	16	33.33%
Infratemporal fossa	18	37.50%
Black turbinate sign	7	14.58%
Masticator muscle involvement	4	8.33%
Mastoiditis	3	6.25%
Nasopharynx, hypopharynx, and soft palate involvement	2	4.17%
Buccal mucosa	1	2.08%
Bone erosions		
Lamina papyracea	8	16.67%
Walls of the maxillary sinus	8	16.67%
Maxilla	3	6.25%
Inferior wall of orbit	2	4.17%
Lesser or greater wing of the sphenoid	2	4.17%
Pterygoid plate	2	4.17%
Nasal septum	2	4.17%
Erosion of the maxilla	2	4.17%
Crista galli	1	2.08%
Wall of the sphenoid sinus	1	2.08%
Lungs		
B/L consolidation	3	6.25%
B/L septal thickening	3	6.25%
B/L minimal pleural effusion	1	2.08%

Extra-Sinus Tissue Involvement

**Figure 2:**
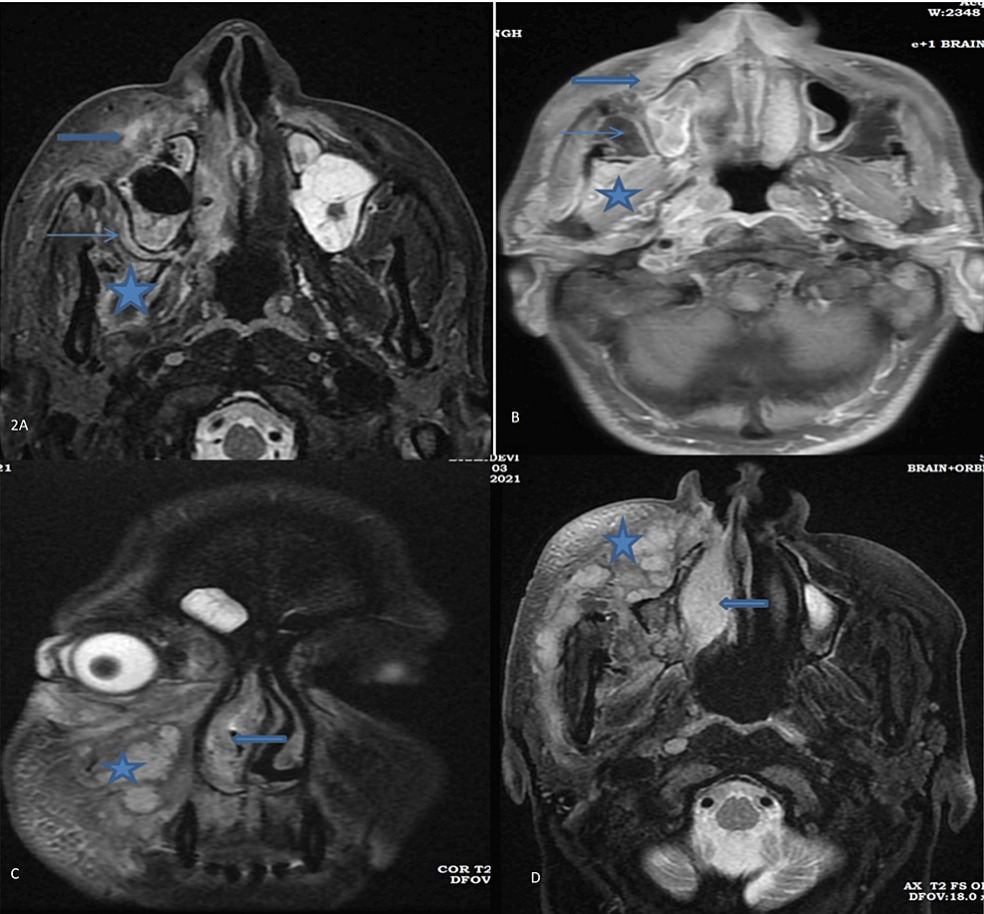
MRI of a 48-year-old male with extension fungal infection in soft tissue around paranasal sinuses. (A) Axial T2WFS reveals hyperintensity in the bilateral (right > left) preantral area (thick arrow) and right retroantral area (thin arrow), along with hyperintense infratemporal muscle (asterisk). (B) Axial T1FS contrast enhancement highlights areas involved in T2FS. (C) Coronal T2FS and (D) axial T2FS demonstrate the extension of infection in the preantral, premaxillary area (asterisk), and hyperintense inferior turbinates (thick arrow).

The most common extra sinus area involved was retroantral fat in 24 (50%) cases. Other involved areas were preantral premaxillary in 23 (47.92%) cases and infratemporal fossa in 18 (37.50%) cases. On imaging, the involved extra-sinus area shows fat stranding, and in severe cases, fluid collection is seen, which appears hyperintense in fat-suppressed imaging and enhanced in T1 fat-suppressed contrast imaging (Figure 2A, 2B, 2C, 2D).

Bone Erosion

**Figure 3:**
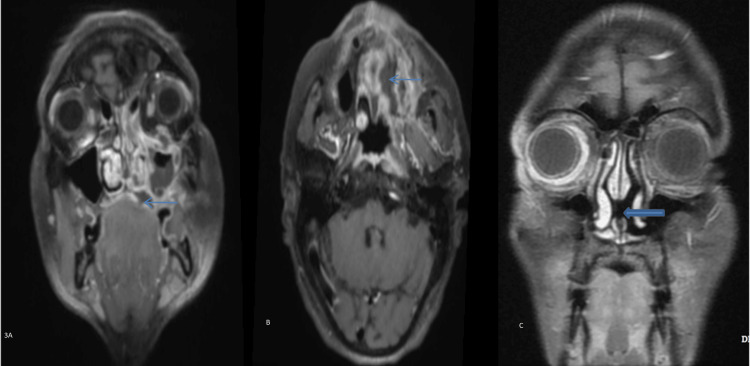
Involvement of the hard palate of a 55-year-old female patient by mucormycosis. (A, B) Coronal and axial T1FS contrast images depict a non-enhanced hypointense area consistent with necrosis surrounded by an enhanced inflamed and infected area in the left maxilla involving the hard palate. (C) Another 22-year-old male exhibits septal perforation on T1FS contrast, as shown in the coronal image (thick arrow).

Erosion, lytic destruction, thinning, or rarefaction were noted in the affected bones. Involvement of the facial bone-like hard palate (Figure 3A, 3B) and maxillary alveolus is seen in severe infection, which shows no enhancement on contrast imaging. Bone destruction of the hard palate can lead to an oroantral fistula, and the maxillary alveolus can cause the loosening of a tooth. Bony septal involvement causes perforation (Figure 3C) in fulminant infections.

The most common bone affected was the wall of the maxillary sinus and lamina papyracea, noted in eight (16.67%) cases, resulting in the spread of nasal infection into the orbit and surrounding areas. Erosion of other bones is mentioned under the heading of bone erosions (Table 2).

There were three patients with features of ROCM along with features of COVID-19 pneumonia. A single case presented with features of COVID-19 pneumonia one day after vaccination with a CT severity score of 12/25 and features of ROCM developed in the second week, discussed under the heading of lungs (Table 2).

Orbit

**Figure 4:**
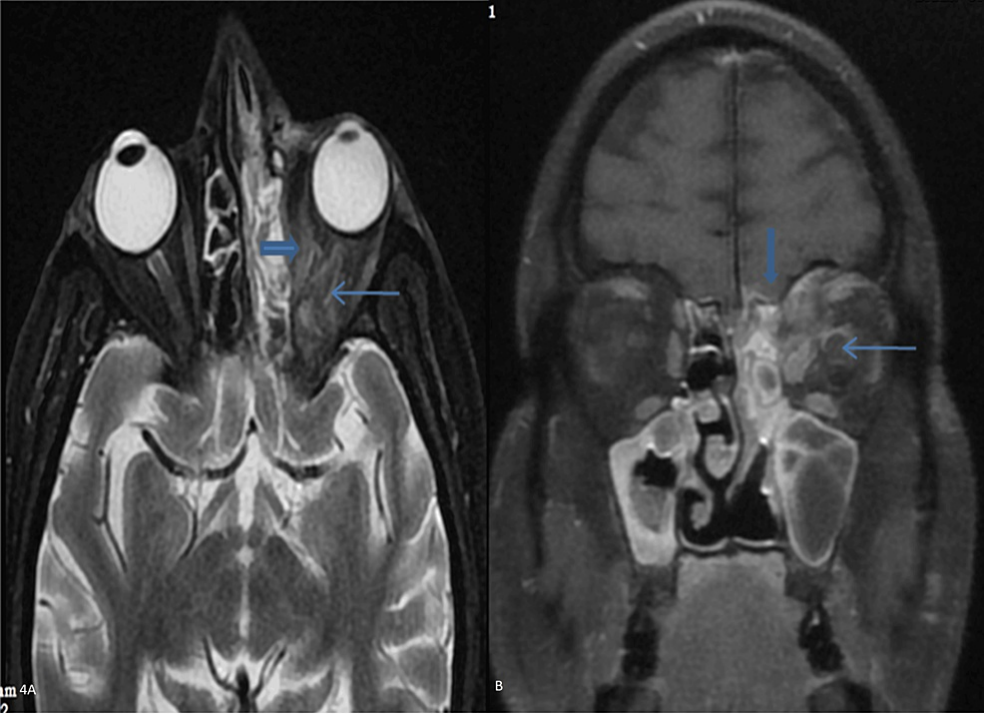
A 52-year-old male patient with retro-orbital fat stranding and optic neuritis in the left orbit. (A) Axial T2FS reveals hyperintense fat stranding (elbow arrow) in the intraconal compartment and hyperintensity along the left optic nerve (thin arrow). (B) The coronal T1FS contrast image displays enhancement of the meninges of the left optic nerve, indicative of optic neuritis, along with enlargement and enhancement of the left medial rectus, superior oblique, and inferior rectus.

Different parts of the orbit and surrounding area were involved in ROCM. A total of 38 (79.17%) cases exhibited involvement of the extraconal compartment, while 32 (66.67%) cases showed involvement of the intraconal compartment. Preseptal cellulitis was noted in 18 (37.50%) cases, which appeared on the CT scan as fat stranding in soft tissue anterior to the orbital septum. ROCM can extend into retro-orbital areas, including both intraconal and extraconal compartments (Figure 4A, 4B).

**Figure 5:**
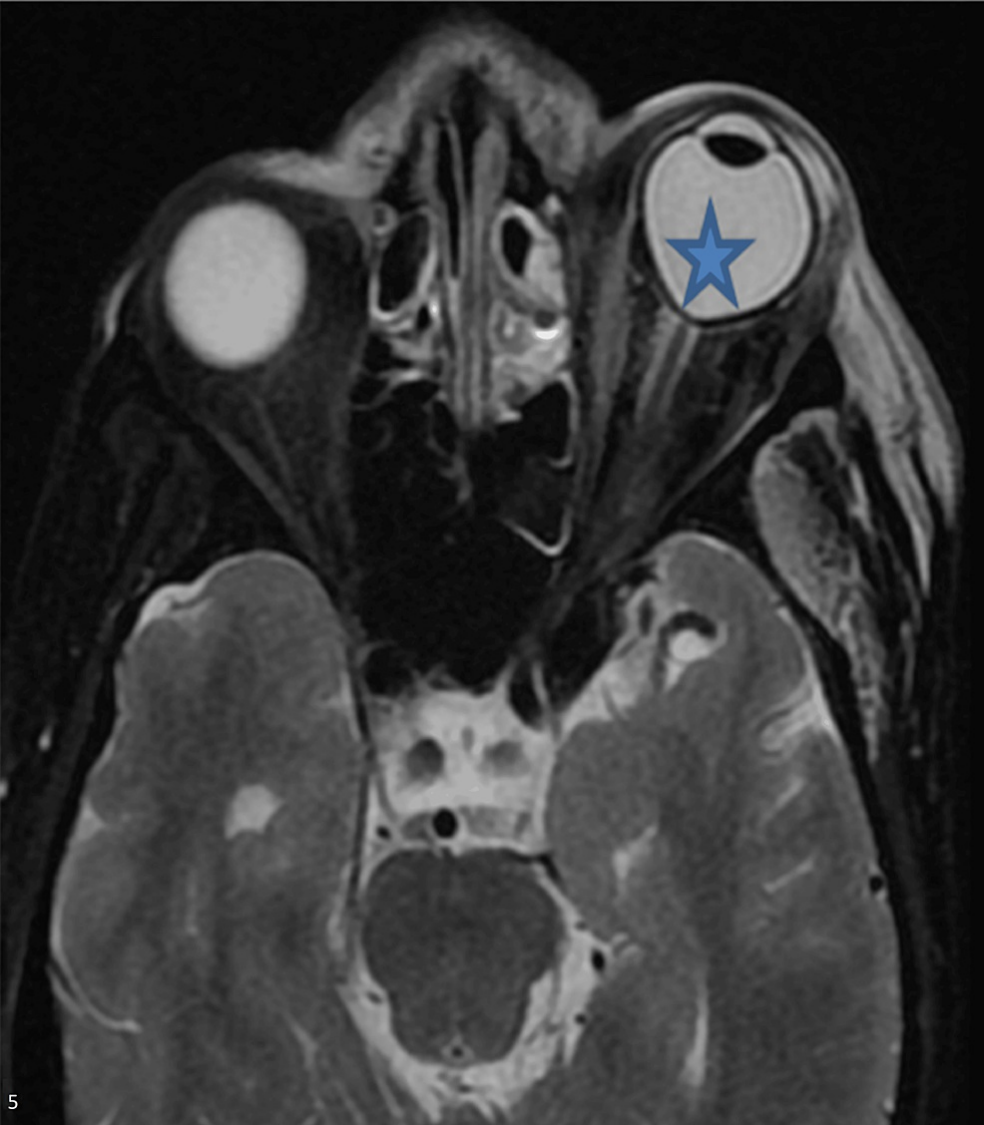
Tenting of the globe. The axial T2W image of the 48-year-old male patient shows a posterior conical shape (asterisk) of the left orbit.

Preseptal cellulitis was seen in 18 (37.50%) cases. In severe infection, the shape of the globe became distorted, known as the “guitar pick sign” or “tenting of the globe” (Figure 5), seen in six (12.50%) cases. Other parts of the involvement of orbit are demonstrated in Table 3.

**Table 3:** Imaging findings of involvement of orbit.

Involvement of parts of the orbit	Frequency	Percentage
Extraconal compartment	38	79.17%
Intraconal compartment	32	66.67%
Preseptal cellulitis	18	37.50%
Extraocular muscle involvement	18	37.50%
Optic nerve involvement	16	33.33%
Orbital apex involvement	9	18.75%
Bilateral orbit	7	14.58%
Distortion or tenting of the globe	6	12.50%
Proptosis	5	10.42%
Right superior ophthalmic vein partial or early thrombosis and retro-orbital extraconal abscess	1	2.08%

A clinical examination of the eye revealed the involvement of many parts of the eye. The most common presentation was chemosis in 28 (58.33%) cases. The rest of the findings of the clinical examination are given in Table 4.

**Table 4:** Distribution of clinical findings from the eye examination.

Eye examination	Frequency	Percentage
Chemosis	28	58.33%
Congestion	25	52.08%
Pupil-dilated, non-reactive, or sluggish reaction	23	47.92%
Ptosis	19	39.58%
Black eschar in the lid or medial canthus	2	4.17%
Corneal haze	2	4.17%
Lagophthalmos	1	2.08%

Intracranial Involvement by Mucormycosis

**Figure 6:**
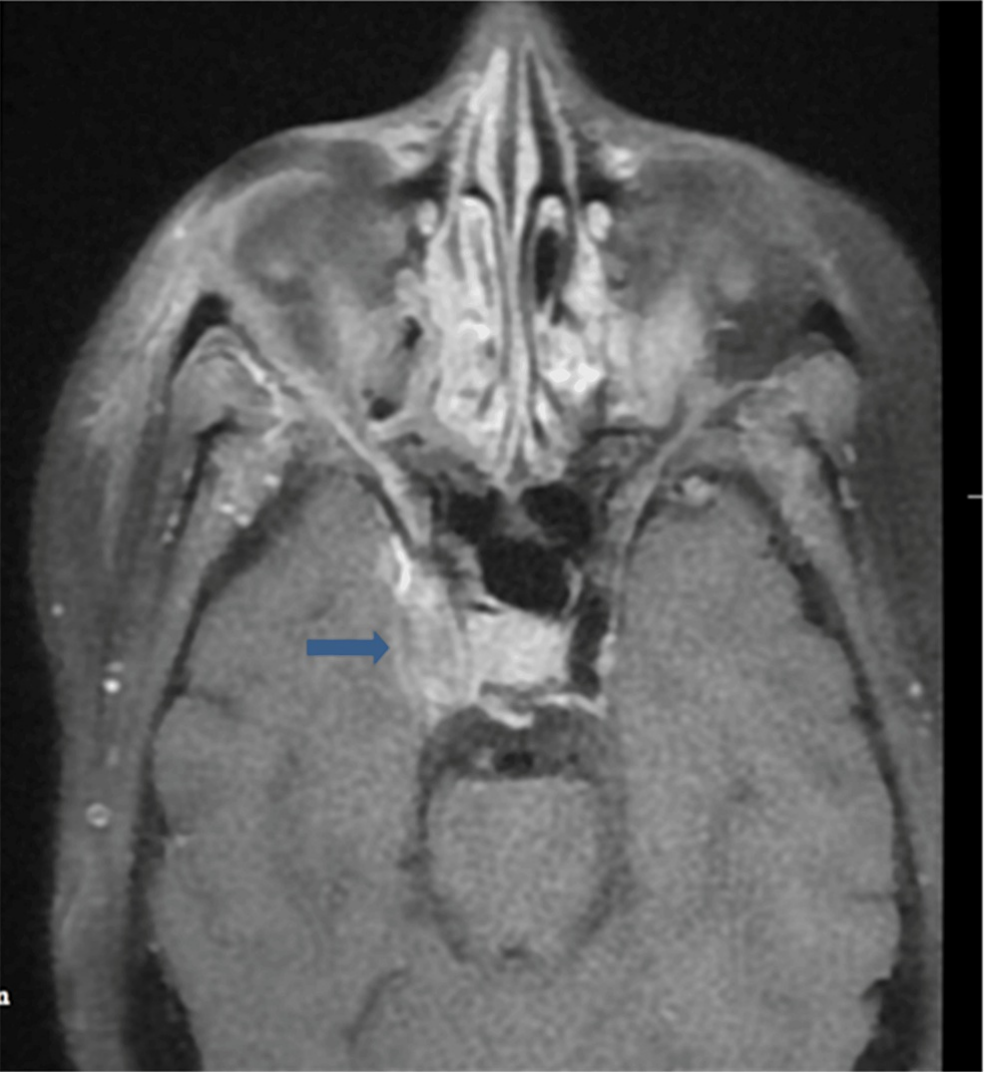
A 40-year-old male patient with right cavernous sinus involvement. Axial T1FS contrast shows enhancement of the right cavernous sinus with loss of signal void in the cavernous part of the internal carotid artery (thick arrow).

Among intracranial involvement, the most common brain infarct was noted in 27% of patients due to the angioinvasive nature of mucormycosis, resulting in the invasion of branches of the internal carotid artery, which appeared to be either loss of signal void or non-enhancement on the postcontrast study. Cavernous sinus involvement was noted in 18.75% of cases, resulting in various orbital manifestations. On imaging, the cavernous sinus shows enhancement on contrast imaging and loss of signal void in or no enhancement of the cavernous segment of the internal carotid artery on contrast imaging (Figure 6).

**Figure 7:**
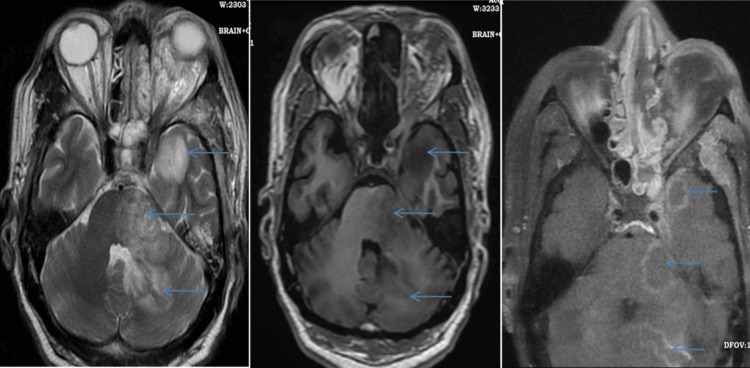
A 50-year-old male patient with intracranial abscesses in mucormycosis. There are areas of hyperintensity on T2W, hypointensity on T1W, and enhancement on contrast study on DWI, which involves the left basal temporal lobe, pons, and cerebellum (multiple thin arrows).

An intracranial fungal abscess was noted in four (8.33%) cases. On imaging, fungal abscesses show edema with a mass effect on surrounding structures and show irregular enhancing walls on contrast imaging (Figure 7) and restriction on DWI, which show suppression on ADC. Other intracranial involvements of MRI findings are given in Table 5.

**Table 5:** MRI findings distribution.

MRI findings	Frequency	Percentage
Infarct in brain parenchyma	13	27%
Cavernous sinus involvement (filling defect or abnormal signal alteration)	9	18.75%
Brain abscess (fungal)	4	8.33%
Encephalitis	3	6.25%
Internal carotid artery and its branch (loss of signal void/non-opacification on contrast)	3	6.25%
Meningitis	2	4.16%
Post-septal extraconal abscess	1	2.08%

Distribution Patterns of Mucormycosis

According to the extension of involvement in mucormycosis, post-COVID-19 rhino-orbital mucormycosis was the most common pattern of involvement. The rest of the patterns of involvement are mentioned in Table 6.

**Table 6:** Distribution of patterns of mucormycosis. ROCM, rhino-orbital-cerebral mucormycosis

Diagnosis	Frequency	Percentage
Post-COVID-19 rhino-orbital mucormycosis	21	43.75%
Post-COVID-19 rhino-orbital cerebral mucormycosis	19	39.58%
Post-COVID-19 rhino mucormycosis	3	6.25%
COVID-19 pneumonia with ROCM	3	6.25%
Post-COVID-19 rhino-cerebral mucormycosis	1	2.08%
Post-COVID-19 orbital mucormycosis	1	2.08%
Total	48	100%

## Discussion

Route of the fungal infection

The fungal infection occurs through the inhalation of fungal spores, which grow in the mucosa of the sinuses and nasal cavity. The spread of infection occurs directly through the foramina of bones, erosion or lysis of bones, invasion of the neurovascular bundle, and lymphatics. Commonly, it spreads outside the sinuses into surrounding tissues by direct extension [7,8].

Common risk factors

Immunocompromised patients, especially diabetic patients, are predominantly affected. Other comorbid conditions are hypertension, hemolymphoid and other malignancies, organ or stem cell transplants, chronic kidney disease, liver disease, lung disease, and heart disease.

The majority of patients developed a fungal infection during the recovery phase of COVID-19 in the present study, as seen in the study of Ashour et al. [2]. In the current study, three cases had features of COVID-19 pneumonia and mucormycosis, but one of the three cases had features of COVID-19 pneumonia after vaccination because the patient had an earlier subclinical infection that showed overt features post-vaccination.

Gender predominance

The male gender was predominantly affected in 70% of cases. Similar trends were found in the United Arab Emirates in a study by Senok et al., as depicted in a systematic review by Seyedjavadi et al. [5]. Sex hormones and X chromosomes play a protective role in innate and adaptive immunity, causing low susceptibility to COVID-19 infection in females [5].

Ecological differences have influenced the occurrence of fungal coinfection. The frequency of fungal coinfection in patients with COVID-19 was higher in Asia than on other continents [5].

Sinonasal involvement

In this study, the ethmoid sinus and maxillary were the most commonly involved sinuses. Unilateral involvement with the sinus involvement of multiple sinuses was more frequently noted by Aggarwal et al. [9], and a similar trend was noted in our study. Sinonasal involvement is seen on CT scans as a mucosal thickening or opacification of involved sinuses, which may be hyperdense due to heavy metal content in fungal hyphae and show heterogeneous or peripheral enhancement. Obliteration of retroantral and periantral fat was an early diagnostic sign of extra-sinusal fungal extension. Fungal sinusitis is differentiated from bacterial or viral sinusitis by bone erosion along with mucosal thickening and high-density content in a fungal infection. In ROCM, bone erosion was seen in the wall of the maxillary sinus, lamina papyracea, and maxilla. On MRI, it appears hypointense on T1W imaging, variable signal intensity on T2W imaging, and variable enhancement on contrast studies, ranging from mild, heterogeneous, intense, and peripheral enhancement. The mentioned angioinvasive strengths include the following: (1) The study provides a comprehensive evaluation of CT and MRI findings in a relatively large cohort of patients with COVID-19-associated ROCM. (2) The detailed description of imaging findings at different stages of the disease has clinical and educational value. The nature of mucormycosis causes necrosis of tissue, resulting in the non-enhancement of T1FS post-contrast study and showing restrictions on DWI. Non-enhancement of nasal turbinates, known as the black turbinate sign, is an early sign of an acute invasive fungal infection. In our study, black turbinate signs were found in seven (14.5%) cases [10].

Bone changes

Bone changes are best visualized on a CT scan. Bone involvement is seen in 40% of cases of mucormycosis [3]. Usually, infection of facial bones, especially the maxilla, in comparison to the mandible is rare due to increased vascularity. Invasion of fungus into cancellous bone causes inflammation and edema, resulting in severe compression of vascularity, leading to ischemia and necrosis of bone. Immobile and stagnant blood is a nidus for infection [11]. Erosion, lysis, and rarefaction are seen in involved bones due to direct or hematogenous invasion by mucormycosis. Common bones involved in our study were the lamina papyracea, the walls of the maxillary sinus, the maxilla, including the maxillary alveolus, palate, and pterygoid plate, and the nasal septum, resulting in septal perforation.

Extrasinus extension

Aggressive fungal infection spreads to the pterygopalatine fossa, infratemporal fossa, and masticator space, which appears as fat stranding and soft tissue thickening on CT and MRI. In the present study, in the case of severe ROCM, the involvement of the soft palate, nasopharynx, and parapharynx was seen in two (4.17%) cases.

Orbital involvement

The most common ocular presentation was chemosis in the current study. Similar findings were seen in a meta-analysis by Bhattacharyya et al. [12]. The medial part of the orbit is involved due to the spread of infection through the lamina papyracea and nasolacrimal duct. Infection may occur in the pterygopalatine fossa through inferior orbital fissures [13]. The medial rectus was most commonly involved due to medial proximity, followed by the inferior rectus. Involved muscles and retrobulbar fat appeared hyperintense on the fluid-sensitive sequence of the MRI. Posterior globe tenting is known as the “guitar pick sign” due to raised intraocular pressure caused by intraorbital inflammation, swelling of extraocular muscles, retro-orbital fat stranding, and focal collection. This sign is also present in other inflammatory conditions and traumatic retrobulbar hemorrhage [14].

Intracranial involvement

The cavernous sinus was the first intracranial structure that was involved due to the angioinvasive nature of mucormycosis. Thrombosis of the cavernous sinus causes cranial nerve (II, IV, ophthalmic, and maxillary branches of V and VI) palsy. Thrombosis of the internal carotid artery and its branches presented with multifocal infarcts that appeared as a restricted area in DWI, a non-enhancement or filling defect on a post-contrast CT scan or MRI, and a loss of signal on a plane MRI. A total of 27% of patients presented with an infarction in the brain and 18.75% with cavernous sinus involvement. The intracranial spread of infection occurs directly through the sinonasal cavity, neurovascular channels like ophthalmic vessels, and the olfactory nerve. Moreover, 8.33% of cases presented with a fungal abscess, which showed restriction on the diffusion-weighted study and peripheral rim enhancement on the postcontrast MRI. Leptomeningeal enhancement was seen in advanced disease by Aggarwal et al. [9], a similar finding noted in the present study in 6.25% of cases. The pathobiology of mucormycosis is characterized by the proliferation of angioinvasive hyphae within the elastic lamina of large to intermediate-sized arteries, which necessitates a meticulous evaluation of the intracranial and facial vasculature [4].

Patterns of involvement

According to imaging, the most common pattern of organ involvement was rhino-orbital mucormycosis (43.7% in the current study, similar to a meta-analysis by Bhattacharyya et al.). According to progression and extent of involvement, we categorized the disease radiologically as Stage 1 rhinosinusitis with no extrasinus extension, Stage 2 rhino-orbital, and Stage 3 ROCM. Similar categorization is done clinically by Firke and Deshmukh [15,16].

Imaging

MRI is more sensitive and has a higher negative predictive value in the detection of early changes in invasive fungal rhinosinusitis due to its exquisite sensitivity for the detection of soft tissue pathology. Orbit involvement, especially orbital apex syndrome, cavernous sinus involvement, and intracranial pathologies, including skull base lesions, are best evaluated by MRI. The main advantage of MRI is the early detection of extrasinus extension, orbital, and vascular invasion.

A CT scan is easily available and is best for the detection of bone changes. It takes less time, and scanning is done in a single breath hold, so usually scans can be done without sedation.

Clinical examination in mucormycosis is inadequate for surgical planning because the area of involvement is not visualized clinically. Imaging, including CT scans and MRI, plays a vital role in early diagnosis, presurgical mapping of the extent of disease, complications, and post-treatment follow-up. Imaging is also important for the assessment of treatment response and the adequacy of surgical debridement. If postoperative follow-up shows residual non-enhancing necrotic areas, this is an indication of re-debridement. There are certain parts of infected areas that are not easily accessible, like the pterygopalatine fossa, which suggests a poor prognosis.

Treatment of ROCM is done through control of blood sugar and surgical debridement of the involved area with systemic antifungal medicine. Liposomal amphotericin B is the drug of choice, with posaconazole as an alternative in cases of intolerance or resistance. The mortality rate for invasive fungal infections is 50-80% [17]. Clinical suspicion of mucormycosis, early diagnosis, and treatment reduce morbidity and mortality. MRI is very helpful in the evaluation of disease extent and neurovascular complications.

Strengths of the study

The study provides a comprehensive evaluation of CT and MRI findings in a relatively large cohort of patients with COVID-19-associated ROCM. The detailed description of imaging findings at different stages of the disease has clinical and educational value.

Limitations of the study

Single-center and retrospective studies are the limitations of this study. All patients did not undergo a contrast study. There is an absence of follow-up imaging to assess the treatment response. The potential selection bias is due to the tertiary care referral setting and the inclusion of only culture- and histopathology-confirmed cases. The multicenter study may overcome the limitations of the present study.

## Conclusions

COVID-19-associated ROCM is an acute, invasive fungal infection with high morbidity and mortality in patients with associated comorbid conditions such as diabetes, treated with steroids, oxygen, and prolonged intensive care support requirements. Early diagnosis and treatment reduce cost, morbidity, and mortality. Imaging with CT and MRI shows disease extent, which provides a road map for surgical debridement, prognosis, post-treatment follow-up, and complications. A CT scan is the modality of choice for detecting bone changes. Radiologists must have a high index of suspicion for early diagnosis and knowledge of the entire imaging spectrum of ROCM. Periantral fat stranding is an early sign that warns radiologists of extra sinus extension of disease, and the non-enhancement of turbinate on postcontrast imaging indicates the invasive nature of mucormycosis. Close attention must be given to extra sinus extension, especially the orbit and brain, to detect early complications.
